# Regulation of I*κ*B*α* Function and NF-*κ*B Signaling: AEBP1 Is a Novel Proinflammatory Mediator in Macrophages

**DOI:** 10.1155/2010/823821

**Published:** 2010-04-12

**Authors:** Amin Majdalawieh, Hyo-Sung Ro

**Affiliations:** ^1^Department of Biology and Chemistry, Faculty of Arts and Sciences, American University of Sharjah, P.O. Box 26666, Sharjah, UAE; ^2^Department of Biochemistry and Molecular Biology, Faculty of Medicine, Sir Charles Tupper Medical Building, Dalhousie University, Halifax, NS, Canada B3H 1X5

## Abstract

NF-*κ*B comprises a family of transcription factors that are critically involved in various inflammatory processes. In this paper, the role of NF-*κ*B in inflammation and atherosclerosis and the regulation of the NF-*κ*B signaling pathway are summarized. The structure, function, and regulation of the NF-*κ*B inhibitors, I*κ*B*α* and I*κ*B*β*, are reviewed. The regulation of NF-*κ*B activity by glucocorticoid receptor (GR) signaling and I*κ*B*α* sumoylation is also discussed. This paper focuses on the recently reported regulatory function that adipocyte enhancer-binding protein 1 (AEBP1) exerts on NF-*κ*B transcriptional activity in macrophages, in which AEBP1 manifests itself as a potent modulator of NF-*κ*B via physical interaction with I*κ*B*α* and a critical mediator of inflammation. Finally, we summarize the regulatory roles that recently identified I*κ*B*α*-interacting proteins play in NF-*κ*B signaling. Based on its proinflammatory roles in macrophages, AEBP1 is anticipated to serve as a therapeutic target towards the treatment of various inflammatory conditions and disorders.

## 1. NF-*κ*B Signaling Pathway

The ability to sense external stimuli that could be lethal to cells coupled with the potential to respond to such cytotoxic signals by switching on defensive genes to sustain cell growth and survival is a remarkable facet of nuclear factor kappa B (NF-*κ*B). Since NF-*κ*B is ubiquitously expressed in almost all types of cells and is a transcription factor that is sequestered in an inactive state in the cytosol but can become activated by a wide range of diverse internal and external stimuli, NF-*κ*B has long been considered an ideal safeguard to defend the cell against countless stimuli and maintain its homeostasis [1]. Moreover, NF-*κ*B is a unique transcription factor in that its function is not solely dependent on its expression when needed. Rather, NF-*κ*B is constitutively expressed in the cell, but it does not become active until it is called upon for action, in which it will be ready and its mission can be accomplished in a timely regulated fashion. 

**Figure 1:**
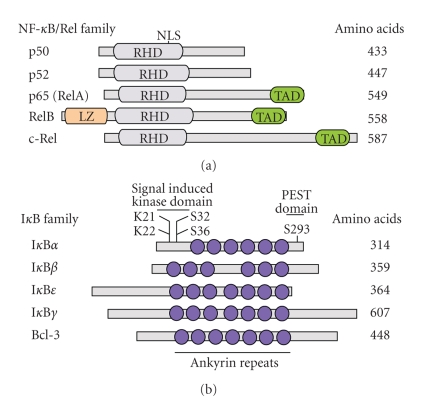
Structural Organization of NF-*κ*B*/*Rel and I*κ*B Proteins. (a) A schematic representation of some members of the Rel family of proteins. Members of this family contain a unique, highly conserved Rel homology domain (RHD) towards the N-terminus, and this domain carries a nuclear localization signal (NLS). Most members of the Rel family contain a C-terminally located transactivation domain (TAD) that is important for optimal transcriptional activity. RelB is a structurally unique member of the NF-*κ*B protein family in that it contains a leucine zipper-like (LZ) region at its N-terminus. (b) A schematic representation of some members of the I*κ*B family of proteins, which are uniquely characterized by the presence of 30-33-amino acid ankyrin (ANK) repeats. At least for I*κ*B*α* and I*κ*B*β* the most well-characterized members of the I*κ*B family, there are two conserved serine residues at the N-terminus preceding the first ANK repeat. Phosphorylation of these two serine residues is known to be crucial for signaling I*κ*B proteins for ubiquitination and proteolytic degradation. At the C-terminus of I*κ*B proteins, there is a region rich with proline, glutamate, serine, and threonine residues, and hence, it is named the PEST domain. The number of amino acid residues within the indicated proteins in mouse is shown.

NF-*κ*B comprises a family of ubiquitously expressed, eukaryotic transcription factors that participate in the regulation of multiple immediate genes that are expressed at the onset of many vital biological processes such as cell growth, immunoregulation, apoptosis, and inflammation [2, 3]. Modulation of NF-*κ*B activity can lead to many abnormal cellular processes and diseases including asthma, arthritis, atherosclerosis, obesity, and various types of cancers [2–7]. NF-*κ*B exists in cells as a heterodimer of members of the Rel family of proteins, including p50, p52, p65 (RelA), RelB, and c-Rel, which share a high degree of structural similarity (Figure 1).

## 2. Roles of NF-*κ*B in Inflammation and Atherosclerosis

One of the major functions of NF-*κ*B is its key involvement in inducing an effective immune/inflammatory response against viral and bacterial infections. The importance of NF-*κ*B role in initiating a potent inflammatory response cannot be better signified than recognizing that the *κ*B consensus sequence is found in the promoter/enhancer regions of more than 50 diverse genes whose expression is known to be crucial in driving an inflammatory response [8–10]. Inducible genes that are known to be transactivated by NF-*κ*B include, but are not limited to, IL-1*β*, IL-6, IL-8, TNF*α*, IFN*γ*, MCP-1, iNOS, COX-2, intracellular adhesion molecule-1 (ICAM-1), and vascular cell adhesion molecule-1 (VCAM-1) [2, 3, 10–19]. These molecules play critical roles in key biological events involving cell recruitment, attachment, differentiation, proliferation, and activation constituting an active inflammatory response. NF-*κ*B is also known to cooperate with other active transcription factors such as activator protein-1 (AP-1) in upregulating the expression of some MMPs [20, 21], which play destructive roles in atherosclerotic lesions rendering them unstable and prone to rupture. 

Genetic knockout models have provided lucid evidence that NF-*κ*B proteins are absolutely essential for the development of a normal, effective immune system, since NF-*κ*B genetic ablation, in general, renders mice immunocompromised and prone to pathogenic infections. Specifically, p50^−/−^ mice develop normally but are defective in immunoglobulin production, and thus, humoral immune responses [22]. Likewise, p52^−/−^ mice develop normally but their B-cell follicles and germinal centers do not develop normally, and the mice are unable to launch an adequate humoral response against T-cell-dependent antigens [23, 24]. Although ablation of p65 causes embryonic lethality due to liver apoptosis [25], ablation of TNF*α* or TNFR rescues p65^−/−^ from the lethal phenotype [26, 27]. However, p65^−/−^/TNF*α* ^−/−^ mice are highly susceptible to bacterial infections and unable to provoke an innate immune response. In addition, T and B lymphocytes of c-Rel^−/−^ mice are unresponsive to various mitogenic stimuli, and the mice are unable to generate a humoral immune response [28]. Lastly, RelB^−/−^ mice are severely defective in generating adaptive immune responses [29]. Thus, it is evident that NF-*κ*B proteins are indispensable in generating effective inflammatory, innate, and adaptive immune responses against viral and bacterial pathogens. 

The first experimental evidence of NF-*κ*B role in atherosclerosis, a progressive inflammatory disease, came from a study demonstrating that active NF-*κ*B can be detected in aortae with evident atherosclerotic lesions but not in normal, nonlesional aortae [30]. In fact, a strong signal of active NF-*κ*B can be detected in endothelial cells, macrophages, and to a lesser extent, T lymphocytes within atherosclerotic lesions [30, 31]. Interestingly, oxLDL is potentially capable of activating NF-*κ*B in endothelial cells and macrophages in culture systems as well as in atherosclerotic lesions [32–35]. In the context of atherosclerosis, NF-*κ*B activation is believed to promote the expression of various factors that mediate various processes such as proliferation, chemotaxis, adhesion, inflammation, and thrombosis, key events in atherogenesis [36]. Wolfrum and colleagues have shown that mice which overexpress TNF-inducible protein A20, a cytosolic zinc finger protein that inhibits NF-*κ*B activity by blocking I*κ*B degradation, display significantly smaller atherosclerotic lesions compared to control mice [37]. A recent study has clearly demonstrated that endothelium-specific inhibition of NF-*κ*B activity is accompanied by significant reduction in atherosclerotic lesion formation in apolipoprotein E null (ApoE^−/−^) mice [38]. In fact, inhibition of NF-*κ*B leads to abrogated macrophage recruitment to the atherosclerotic lesions and reduced expression of cytokines and chemokines in the aortae of ApoE^−/−^ mice [38]. Indeed, a large number of naturally occurring products have been shown to attenuate the pathogenesis of atherosclerosis by virtue of their ability to interfere with NF-*κ*B signaling [39–43]. Furthermore, several studies have demonstrated a positive correlation between NF-*κ*B activity and incidence of myocardial infarction [44–51]. Due to its critical role in atherosclerosis and myocardial infarction, NF-*κ*B is proposed to be a promising therapeutic target for reducing, if not eliminating, the risks of atherosclerosis and its complications.

## 3. Structure of NF-*κ*B/Rel Proteins

Although several homodimers and heterodimers are formed by various members of the NF-*κ*B protein family, NF-*κ*B is a term that is often used to describe the p50/p65 heterodimer, which was the first NF-*κ*B dimer to be described [18, 52]. Indeed, p50 and p65 are the first members of the NF-*κ*B gene family to be cloned and characterized [53–56]. As shown in Figure 1, members of the NF-*κ*B/Rel protein family contain a highly conserved, N-terminal 300-amino acid region known as the rel homology domain (RHD), which mediates dimerization, interaction with I*κ*B proteins, nuclear translocation due to the presence of a nuclear localization signal (NLS) within RHD, as well as binding to specific sites within the promoters of target genes [10]. Although the majority of NF-*κ*B dimers are capable of transactivating target genes, in vivo data demonstrated that some dimers such as p50/p50 and p52/p52 homodimers can be inactive or repressive [57–59]. The fact that p50 and p52 lack a C-terminal region that is conserved in the majority of other NF-*κ*B proteins suggests that this region confers on NF-*κ*B proteins a transcriptional potential, and hence, it is called the transactivation domain (TAD) [10]. Mutations of important residues within TAD render activating NF-*κ*B dimers transcriptionally inactive [60]. RelB is a structurally unique member of the NF-*κ*B protein family in that it contains a leucine zipper-like (LZ) region at its N-terminus, which is required for its full transcriptional activity [61].

## 4. Regulation and Activity of NF-*κ*B/Rel Proteins

Under basal conditions, most NF-*κ*B subunits are sequestered in the cytosol, where they are constitutively bound by members of the NF-*κ*B inhibitor family of proteins, mainly I*κ*B*α* and I*κ*B*β* [54, 62]. However, diverse stimuli including inflammatory cytokines, mitogens, lipopolysaccharides, UV light, as well as bacterial and viral pathogens can transduce a signal that ultimately results in NF-*κ*B liberation from its inhibitors, allowing NF-*κ*B dimers to translocate to the nucleus and become transcriptionally active [9, 63, 64]. Except for RelB, all other NF-*κ*B proteins contain a protein kinase A (PKA) phosphorylation site 20–30 amino acids N-terminal of the NLS within RHD, and phosphorylation of this site (S^337^ in p50 and S^276^ in p65) seems to be essential for nuclear translocation [65, 66]. Moreover, phosphorylation of the PKA phosphorylation site is important in protecting the transcriptional and DNA-binding activities of active NF-*κ*B dimers [67–70]. Studies have shown that S^276^ in p65 is a major phosphorylation site that is subject to compartment-specific and stimulus-specific phosphorylation by PKAc in the cytoplasm [70] and the mitogen- and stress-activated protein kinase-1 (MSK1) in the nucleus [71]. S^276^ phosphorylation is required for optimal NF-*κ*B activity in different cell types [71–73]. Other important phosphorylation sites have been demonstrated to be critical for optimal NF-*κ*B transactivation potential. Such sites include protein kinase C zeta-(PKC*ζ*-) phosphorylated S^311^ [74], casein kinase II (CKII)-phosphorylated S^529^ [75], IKK*α* */* *β*-phosphorylated S^536^ [76–78]. S^536^ in p65 has also been shown to be subject to phosphorylation by other kinases such as AKT/protein kinase B (PKB) [79, 80], ribosomal S6 kinase 1 (RSK1) [81], TRAF family member-associated (TANK)-binding kinase 1 (TBK1) [82, 83], and IKK*ε* [83] under certain circumstances. 

Dimerization of NF-*κ*B proteins is a prerequisite for NF-*κ*B to become transcriptionally active, and it is mediated by specific motifs within RHDs of both members of NF-*κ*B dimers [9]. Studies have shown that the dimerization motifs are located at the C-terminus of RHD, and mutation of critical residues within such motifs interferes with dimerization [84–86]. Site-directed mutagenesis experiments also revealed the importance of certain residues within the dimerization motifs in determining partner specificity [85–87]. Once in the nucleus, active NF-*κ*B dimers can bind to specific DNA-binding sites, known as *κ*B binding sites, within the regulatory regions of their target genes, leading to gene transactivation [10, 88]. The *κ*B site has a conserved consensus sequence of 10 nucleotides (GGGRNNYYCC where N is any base, R is a purine, and Y is a pyrimidine), and slight variations of the *κ*B nucleotide sequence confer preference to different dimer combinations of NF-*κ*B subunits [9, 88]. The N-terminus of RHD is known to be essential for DNA-binding activity of NF-*κ*B proteins [84, 89]. Although point mutations of specific residues within this region do not interfere with dimerization, they completely abrogate the DNA-binding activity of NF-*κ*B dimers [84, 90].

## 5. Inhibitors of NF-*κ*B

Work from Baltimore's laboratory provided initial characterization of NF-*κ*B coordinate regulation via physical interaction with its inhibitors, members of the I*κ*B family of proteins [8, 91]. The observation that nuclear NF-*κ*B exists in an I*κ*B-unbound state indicated that I*κ*B proteins can sequester NF-*κ*B in an inactive state in the cytosol. Initial characterization of I*κ*B proteins that associate with NF-*κ*B led to the identification of 37-kDa and 43-kDa proteins, which are now known as I*κ*B*α* and I*κ*B*β*, respectively [92]. I*κ*B*α* and I*κ*B*β* are the most well-characterized members of the mammalian I*κ*B family of proteins, which contains a number of structurally related proteins besides I*κ*B*α* and I*κ*B*β*, including I*κ*B*γ* *1*, I*κ*B*γ* *2*, I*κ*B*δ*, I*κ*B*ε*, I*κ*BR, I*κ*BL, p100, p105, and Bcl-3 [9, 93]. Recently, a new member of the I*κ*B protein family was identified and named I*κ*B*ζ* [94]. Except for Bcl-3 and I*κ*B*ζ*, which are constitutively localized in the nucleus [94, 95], all other I*κ*B proteins are localized in the cytosol [93]. Nuclear localization of Bcl-3 and I*κ*B*ζ* indicates that these proteins do not regulate NF-*κ*B translocation into the nucleus, but rather, they seem to be involved in regulating NF-*κ*B transcriptional and DNA-binding activities [94, 96–98].

## 6. Structure of I*κ*B*α* and I*κ*B*β*

Structural organization of I*κ*B proteins started to be uncovered upon molecular cloning and characterization of the I*κ*B*α* gene (also known as MAD-3) in the early 1990s [99, 100]. Now, it is clear that all I*κ*B proteins known to date possess three to seven centrally located, 30–33 amino acid repeated sequences known as ankyrin (ANK) repeats (also known as notch-related motifs, cell cycle repeats, and cdc10/SW16 repeats) (Figure 1) [9, 93, 101]. These repeats were initially identified in the SW16 protein expressed by *Saccharomyces cerevisiae* [102]. Although the exact amino acid sequences of ANK repeats found in different I*κ*B proteins can be distinct, ANK repeats have a consensus amino acid sequence (XGXTPLHLAARXGHVEVVKLLLDXGADVNAXTK, where X can be any amino acid) [103, 104]. Even within the same I*κ*B protein, ANK repeats can be quite distinct, and this is thought to be an important determinant in the specificity and selectivity of the protein-protein interaction between I*κ*B and NF-*κ*B proteins [105]. The presence of ANK repeats in I*κ*B proteins renders them capable of physically interacting with regions within the RHD of target NF-*κ*B dimers [106–108]. Additionally, I*κ*B*α*, I*κ*B*β*, and I*κ*B*ε* have N-terminal signal-receiving domain (SRD) containing two highly conserved serine residues, which are known to be important phosphorylation sites involved in the regulation of I*κ*B function [9, 93]. I*κ*B*α*, I*κ*B*β*, I*κ*B*γ* *1*, I*κ*B*γ* *2*, I*κ*B*δ*, I*κ*BR, I*κ*BL, p100, and p105 contain a region at the C-terminus that is rich in proline, glutamate, aspartate, serine, and threonine residues, and hence, it is called the PEST domain [9, 93]. The PEST domain plays an important role in the inhibition of NF-*κ*B DNA-binding activity [109], as well as in I*κ*B protein stability/turnover [52, 110–113]. Although deletion of the N-terminus and/or the C-terminus does not affect I*κ*B*α* ability to interact with NF-*κ*B dimers, point mutations of certain residues within the N-terminus of I*κ*B*α* render it resistant to signal-induced phosphorylation and degradation [114–117], while deletion of the C-terminus of I*κ*B*α* interferes with its ability to dissociate NF-*κ*B from its DNA binding sites [107, 109, 118]. Finally, two nuclear export signal (NES) sequences have been identified in the N-terminus [119] and C-terminus of I*κ*B*α* [120]. Actually, the more conserved N-terminal NES was shown to be necessary and sufficient for I*κ*B*α* nuclear export [119]. Efficient nuclear translocation and cytosolic relocalization (i.e., nuclear export) of I*κ*B*α* is ensured by the presence of NLS and NES, respectively.

## 7. Function of I*κ*B*α* and I*κ*B*β*

Since I*κ*B*α* and I*κ*B*β* are the best studied members of the I*κ*B protein family, special emphasis will be allotted to these two molecules throughout this paper. Members of the I*κ*B protein family are constitutively and ubiquitously expressed proteins that localize in the cytosol, except for Bcl-3 and I*κ*B*ζ* which are primarily present in the nucleus [94, 95]. The main function of I*κ*B proteins is to inhibit NF-*κ*B activity when it is not required, and this happens via protein-protein interaction that takes place between I*κ*B proteins and NF-*κ*B dimers in the cytosol. I*κ*B*α* and I*κ*B*β* interact via their ANK repeats with the RHD of NF-*κ*B dimerized proteins in such a way that masks the positively charged regions of the NLSs within the RHDs of NF-*κ*B dimers [121, 122]. As a result, NF-*κ*B dimers are prevented from translocating to the nucleus, and thus, they are kept in an inactive, I*κ*B-bound state in the cytosol [123–125]. Although I*κ*B-NF-*κ*B interaction is mediated by ANK repeats of I*κ*B proteins, not all ANK repeats are involved in this interaction [107, 118, 126]. In an extensive site-directed mutagenesis study performed to assess the significance of every ANK repeat within I*κ*B*α* [107], a number of interesting findings were revealed. First, the C-terminus of I*κ*B*α* is required for the protein to be functional, and thus, the ANK repeats are not sufficient on their own to exert an inhibitory action towards NF-*κ*B. Second, lack of the third ANK repeat does not impede I*κ*B*α* inhibitory function, suggesting that this ANK repeat is dispensable for I*κ*B*α* inhibitory function. Third, the only mutant forms of I*κ*B*α* that are unable to inhibit NF-*κ*B activity are those that were incapable of interacting with NF-*κ*B. Another study suggests that the first ANK repeat of I*κ*B*α* is mostly responsible for its inhibitory activity, and substituting the first ANK repeat in I*κ*B*β* with that of I*κ*B*α* significantly enhances the former's inhibitory activity [127]. It is evident that the ANK repeats and the C-terminal region (i.e., PEST domain) of I*κ*B*α* form a tertiary structure that is capable of interacting with NF-*κ*B proteins, and that such interaction confers NF-*κ*B transcriptional inactivity [107].

It is known that NF-*κ*B is itself an upregulator of I*κ*B*α* and I*κ*B*β*, in which NF-*κ*B activation via various and distinct stimuli is usually followed by rapid induction of I*κ*B*α* and I*κ*B*β* expression [19, 52, 128] due to the presence of a *κ*B DNA-binding site within the I*κ*B promoter [129–131]. This negative feedback regulatory loop sets a molecular switch that ensures rapid, controlled, and transient activation of target genes by NF-*κ*B. Induced expression of I*κ*B*α* allows translocation of nascently synthesized I*κ*B*α* into the nucleus, where it binds to active NF-*κ*B dimers that are bound to *κ*B sites within the promoters of their target genes. Interaction between nuclear I*κ*B*α* and active NF-*κ*B dimers leads to dissociation between NF-*κ*B dimers and DNA, and it forces a conformational change in I*κ*B*α* that exposes the nuclear export signal (NES), eventually leading to resequesterization of I*κ*B*α* and NF-*κ*B dimers in the cytosol [92, 120, 132]. This highly complex, tightly regulated reciprocal regulatory process involving NF-*κ*B and I*κ*B proteins confers the NF-*κ*B signaling pathway a central regulatory function in many key biological events that requires transient, short-term NF-*κ*B activity.

## 8. Regulation of I*κ*B*α* and I*κ*B*β*

**Figure 2:**
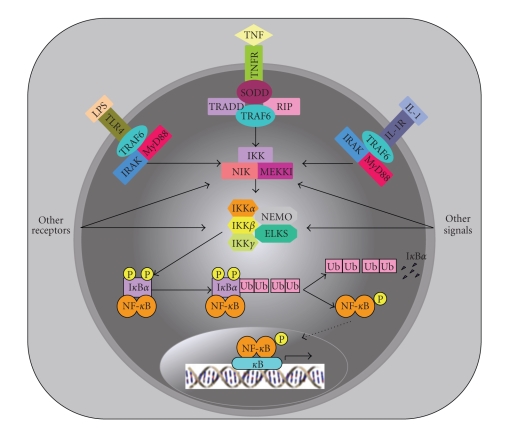
NF-*κ*B Signaling Pathway. A cartoon representing the cascade of biochemical events that are initiated by various stimuli, eventually leading to NF-*κ*B nuclear translocation and transcriptional activation. Recruitment of different adaptor molecules to different receptor complexes coupled with activation of different downstream kinases is shown. There are mainly two signaling pathways leading to NF-*κ*B activation, classical (also known as canonical) and alternative. The formation and activation of the IKK complex, which consists of catalytically active kinases (e.g., IKK*α*, IKK*β*, and IKK*γ*) and noncatalytic regulatory proteins (e.g., NEMO and ELKS), is a universal event in both signaling pathways. In the classical signaling pathway, ligand binding to a cell surface receptor leads to the recruitment of adaptor proteins (e.g., TRAF6) to the receptor, leading to the recruitment of IKK complex and subsequent phosphorylation and degradation of the I*κ*B proteins. Unlike the classical signaling pathway, the alternative signaling pathway, which is normally triggered by non-proinflammatory cytokines (e.g., LT*β*, BAFF, and CD40L) as well as some viruses (e.g., HTLV and EBV), does not allow the recruitment of NEMO. Instead, ligand binding to a cell surface receptor leads to the recruitment of NIK, which in turn phosphorylates and activates IKK*α* dimers. Typically, the classical signaling pathway leads to the activation of NF-*κ*B dimers consisting of RelA, c-Rel, RelB, and p50, while the alternative signaling pathway leads to the activation of NF-*κ*B dimers consisting primarily of RelB and p52.

There are at least two well-characterized signaling pathways leading to NF-*κ*B activation, classical and alternative, and both rely on the catalytic activity of known I*κ*B kinases (IKKs) (Figure 2). The classical NF-*κ*B signaling pathway is typically triggered by a vast number of proinflammatory cytokines (e.g., IL-1*β* and TNF*α*), viruses, and bacteria, and hence, it leads to a coordinate inflammatory/immune response culminating in the expression of multiple cytokines, chemokines, adhesion molecules, and proinflammatory proteolytic enzymes [133, 134]. On the other hand, the alternative NF-*κ*B signaling pathway is normally triggered by non-proinflammatory cytokines (e.g., lymphotoxin *β* (LT*β*), B-cell activating factor (BAFF), and CD40 ligand (CD40L)) as well as some viruses (e.g., human T-cell leukemia virus (HTLV) and Epstein-Barr virus (EBV)) [133, 134]. The alternative NF-*κ*B signaling pathway is triggered to induce the expression of genes whose products play fundamental roles in the development and maintenance of secondary lymphoid organs [133]. Unlike the classical pathway, the alternative pathway is NEMO independent in that it does not require the I*κ*B kinase (IKK) complex, which contains the scaffold protein NF-*κ*B essential modulator (NEMO), IKK*α*, IKK*β*, IKK*γ*, and other adaptor proteins [133–140]. Instead, the alternative pathway relies on the activity of the NF-*κ*B inducing kinase (NIK) that transactivates IKK*α*-IKK*α* homodimers, which upon activation transduce a signal that culminates in profound NF-*κ*B activation [134]. In the next sections, special attention will be paid to the classical NF-*κ*B signaling pathway.

## 9. Basal Turnover/Degradation of I*κ*B*α* and I*κ*B*β*

Besides signal-induced proteolytic degradation of I*κ*B*α* and I*κ*B*β*, these proteins have been shown to be susceptible to degradation under basal, unstimulatory conditions. In fact, I*κ*B*α* and I*κ*B*β* have been shown to be constitutively phosphorylated in absence of stimuli [67, 141], and specific serine/threonine residues (S^283^, S^289^, S^293^, and T^291^) within the PEST domain of I*κ*B*α* have been shown to be the target of constitutive phosphorylation by CKII [111, 113, 117, 142]. Phosphorylation of the PEST domain renders I*κ*B*α* susceptible to degradation, indicating that the PEST domain is essential for controlling I*κ*B*α* intrinsic protein stability [111–113]. Likewise, it was shown that the PEST domain of I*κ*B*β* is required for its degradation [143]. Unlike signal-induced degradation of I*κ*B*α*, which required ubiquitination, basal degradation of I*κ*B*α* seems to be ubiquitination independent, in which degradation of unubiquitinated I*κ*B*α* is evident in unstimulated cells in vitro [144]. This data is supported by the observation that a mutant form of I*κ*B*α* carrying lysine-to-arginine substitutions at the two ubiquitination sites (K^21^ and K^22^) is as prone to basal degradation as the wild type form (WT) of I*κ*B*α* [145]. In other words, ubiquitination of I*κ*B*α* is a signal-induced event and is not required for basal degradation of I*κ*B*α*. However, the ubiquitin-independent I*κ*B*α* degradation pathway is proteasome dependent, since proteasome inhibitors block basal, as well as signal-induced, degradation of I*κ*B*α* [144]. 

Until the emergence of a paper published by Phillips and Ghosh in 1997 [146], the 26S proteasome-mediated proteolysis pathway was the only known cellular process responsible for basal and signal-induced degradation of I*κ*B*α* and I*κ*B*β*. However, the use of selective proteasome inhibitors revealed the existence of a novel proteolysis pathway that leads to I*κ*B*α* and I*κ*B*β* degradation in an ubiquitin-independent, proteasome-independent manner in immature B cells [146, 147]. Indeed, such a novel pathway was subsequently shown to be dependent on the presence of free calcium, most likely imported from outside the cell [145]. Further examination of this pathway revealed that phosphorylation of the PEST domain of I*κ*B*α* allows it to bind to the calmodulin-like domain (CaMLD) of the large subunit of the calcium-dependent thiol protease complex, calpain [148, 149]. Interaction between I*κ*B*α* and calpain is followed by N-terminal cleavage and further proteolysis of I*κ*B*α* [148, 149]. These studies suggest that I*κ*B*α* and I*κ*B*β* can also be regulated by protease machineries other than the intrinsic, well-known 26S proteasome complex.

## 10. Signal-Induced I*κ*B*α* and I*κ*B*β*Phosphorylation by the IKK Complex

In order for NF-*κ*B to become activated, I*κ*B*α*/*β* must become phosphorylated at specific serine residues at the N-terminus, followed by ubiquitination (not for I*κ*B*β*) and proteolytic degradation of phosphorylated I*κ*B*α* and I*κ*B*β* in the cytosol. Phosphorylation and subsequent proteolytic degradation of I*κ*B*α* and I*κ*B*β* liberate NF-*κ*B dimers, which become phosphorylated, translocate to the nucleus, and bind to specific *κ*B binding sites within the promoter/enhancer regions of their target genes, leading to their transactivation. Binding of TNF*α* to its receptor (TNFR1) is known to trigger NF-*κ*B activation through the classical pathway, leading to TNF-induced cell death [150]. Under basal conditions, constitutive activation of the TNF-induced cell death pathway is prevented by the blocking potential of a protein called the silencer of death domains (SODDs), which binds to TNFR1 and prevents downstream signal transduction [150]. Upon TNF*α*-TNFR1 binding, SODD dissociates from TNFR1 and this allows recruitment of adaptor molecules TNF receptor-associated death domain (TRADD), receptor interacting protein (RIP), and TNFR associated factor 2 (TRAF2), which bind to TNFR1 as a complex through TRADD. Sequential recruitment of NIK and the IKK complex to the TRADD complex bound to TNFR1 is mediated by TRAF2 [151, 152]. Stimulation signals triggered by LPS, IL-1*β*, and TNF*α* also lead to the recruitment and activation of MEKK1 [153]. The recruited IKK complex also contains an I*κ*B kinase regulatory subunit called ELKS (glutamic acid, leucine, lysine, and serine-rich protein), which allows I*κ*B*α* recruitment and interaction with the IKK complex at the membrane [154]. Although NEMO, IKK*α*, IKK*β*, IKK*γ*, and ELKS are the main components of the cytoplasmic serine-protein-kinase multi-subunit IKK complex, other proteins are identified as essential elements of the complex [135–140, 155–157]. NIK and MEKK1 are upstream upregulators of the IKK complex, in which they phosphorylate and transactivate IKK*α* and IKK*β* within the complex [155, 158].

Membrane recruited IKK complex with catalytically active IKK*α* and IKK*β* is responsible for phosphorylating two N-terminally located conserved serine residues in I*κ*B*α* and I*κ*B*β* (Ser^32^ and Ser^36^ in I*κ*B*α*; Ser^19^ and Ser^23^ in I*κ*B*β*) [137, 159]. Interestingly, cell lines that lack NEMO display severe defects in NF-*κ*B activation and they are unresponsive to a wide range of potent stimuli [139], indicating that catalytically active IKK*α* and IKK*β* are insufficient in phosphorylating I*κ*B*α* and I*κ*B*β* in the absence of complex formation. Although some studies have initially suggested a major role of IKK*α* in I*κ*B*α* and I*κ*B*β* phosphorylation [135, 137, 156, 160, 161], a study demonstrated that mutation of two serine residues within the activation loop of IKK*β*, but not IKK*α*, renders the IKK complex catalytically inactive [162], indicating that IKK*β* is the predominant kinase component of the IKK complex. This observation is supported by an experiment demonstrating that IKK*α* ^−/−^ cells display normal IKK activity towards I*κ*B*α* and I*κ*B*β* upon LPS, IL-1*β*, and TNF*α* treatment [163]. Moreover, IKK*β* ^−/−^ mice resemble p65^−/−^ mice in that they suffer from embryonic lethality due to severe liver apoptosis [164]. The apoptotic phenotype of IKK*β* ^−/−^ mice combined with the observation that TNF*α* deficiency eliminate embryonic lethality of p65^−/−^ mice [165] and that NF-*κ*B mediates TNF*α*-induced apoptosis [166] strongly suggest that IKK*β* catalytic activity is absolutely required for NF-*κ*B activation. Direct experimental evidence indicates that IKK*β* ^−/−^ embryonic stem cells and fibroblasts display defective IKK activity towards I*κ*B*α*, and no NF-*κ*B activity [164]. These findings indicate that IKK*α* cannot compensate for IKK*β* loss, and that IKK*β* is solely responsible for phosphorylating I*κ*B*α* and I*κ*B*β* in vivo. Interestingly, phosphorylation of S^32^/S^36^ and S^19^/S^23^ in I*κ*B*α* and I*κ*B*β*, respectively, does not force I*κ*B*α* and I*κ*B*β* dissociation from their NF-*κ*B dimer partners in the cytosol [116, 167], but it renders them susceptible to ubiquitination and subsequent proteolytic degradation [101, 167–171].

## 11. Signal-Induced Degradation of I*κ*B*α* and I*κ*B*β*

**Figure 3:**
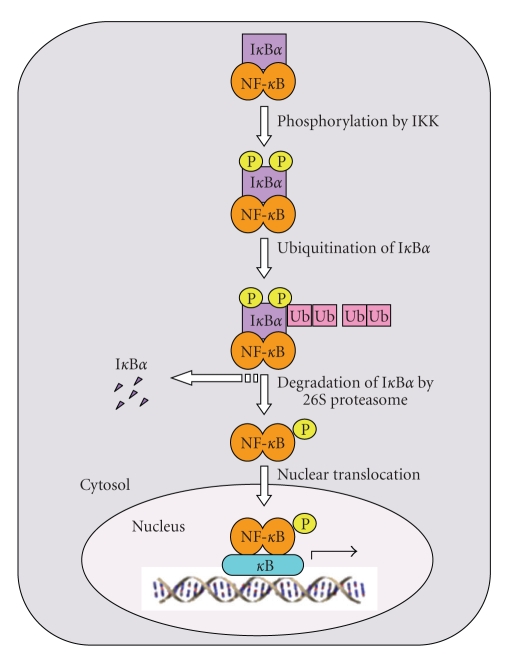
Regulation of I*κ*B*α* and I*κ*B*β*. A cartoon representing the most critical intracellular events leading to NF-*κ*B transcriptional activation. Interaction between diverse ligands and their receptors eventually leads to activation of the IKK complex, which allows I*κ*B*α* and I*κ*B*β* phosphorylation in the cytosol. This phosphorylation step is followed by I*κ*B*α*, but not I*κ*B*β*, ubiquitination, and subsequently, I*κ*B*α* and I*κ*B*β* are subjected to proteolytic degradation. Once I*κ*B*α* and I*κ*B*β* molecules are degraded, NF-*κ*B dimers are liberated, and they translocate to the nucleus subsequent to unmasking of their NLS. Once in the nucleus, NF-*κ*B dimers bind to *κ*B sites within the promoter/enhancer regions of their target genes, driving gene transactivation.

Proteolysis, or proteolytic degradation, is a highly regulated cellular multistep process that involves enzymes called proteases that are capable of hydrolyzing peptide bonds within polypeptides that are usually ubiquitinated, ultimately leading to protein degradation (Figure 3). Protein ubiquitination and subsequent degradation were thought to be molecular mechanisms undertaken by cells to eradicate misfolded or defective proteins [172, 173]. Nevertheless, it is well recognized that protein degradation is a process that is not directed only against imperfect proteins, but also against some fully functional proteins as a means to regulate and control various key biological processes [10]. Cyclins, proteins involved in the regulation of the cell cycle, are a prime example of functional proteins that are regulated by ubiquitination-dependent proteolytic degradation pathways [174, 175]. 

It is evident that signal-induced phosphorylation of I*κ*B*α* and I*κ*B*β* must be followed by their degradation for NF-*κ*B transactivation potential to be manifested [167, 176–182]. The most compelling evidence indicating the necessity of I*κ*B*α* and I*κ*B*β* degradation for NF-*κ*B activation came from a study showing that treatment of stimulated cells with protease inhibitors does not eliminate phosphorylation of I*κ*B*α* and I*κ*B*β*, but protects NF-*κ*B-I*κ*B*α* and NF-*κ*B-I*κ*B*β* cytosolic complexes, and thus, prevents NF-*κ*B activation [143, 167, 178, 181, 182]. Under stimulatory conditions, proteolytic degradation of I*κ*B*α* and I*κ*B*β* occurs via an ubiquitination- and proteasome-dependent mechanism [169, 170].

The 26S proteasome is composed of a core protease, known as the 20S proteasome, and the 19S regulatory complex (RC), which is composed of at least 18 different subunits in two subcomplexes known as the lid and the base [183]. The involvement of the 26S proteasome in signal-induced NF-*κ*B activation was originally signified by studies demonstrating that treatment with selective inhibitors of the 26S proteasome blocks NF-*κ*B activation [178, 184]. Subsequent to phosphorylation of the two serine residues within the signal-induced kinase domain of I*κ*B*α*, multiple 76-amino acid ubiquitin polypeptides covalently attach to the N-terminus of phosphorylated I*κ*B*α*, rendering I*κ*B proteins susceptible to 26S proteasome-dependent degradation [169, 170, 185, 186]. For I*κ*B*α*, ubiquitination primarily takes place on two adjacent lysine residues (K^21^ and K^22^) in the N-terminus of the protein, and mutation of these two lysine residues prevents I*κ*B*α* ubiquitination and subsequent proteolytic degradation [187, 188]. Indeed, conservative substitution of K^21^ and/or K^22^ by arginine precludes not only ubiquitination, but also signal-induced degradation of I*κ*B*α*, ultimately preventing NF-*κ*B activation [187, 188]. During initial characterization of I*κ*B*β* regulation, it was shown that treatment of cells with protease inhibitors prevents I*κ*B*β* degradation [143], suggesting that I*κ*B*β* may be under control of the ubiquitin-proteasome machinery in a phosphorylation-dependent fashion, as in I*κ*B*α*. Indeed, site-directed mutagenesis of S^19^ and/or S^23^ renders I*κ*B*β* somewhat resistant to degradation [143]. Strikingly, however, alanine substitution of K^9^ has no effect on I*κ*B*β* degradation [143], indicating that ubiquitination is not a prerequisite for I*κ*B*β* degradation. So, although phosphorylation of the two, N-terminal conserved serine residues is required for inducing I*κ*B*α* and I*κ*B*β* degradation, ubiquitination of the N-terminal lysine residues is required for proteasome-dependent degradation of I*κ*B*α*, but not I*κ*B*β*. Interestingly, although the PEST domain of I*κ*B*α* and I*κ*B*β* is not required for S^32^/S^36^ and S^19^/S^23^ phosphorylation, respectively, its deletion eliminates signal-induced degradation of I*κ*B*α* [115, 117, 189–191] and I*κ*B*β* [192–194]. In sum, for signal-induced NF-*κ*B transactivation activity to manifest, at least six main biochemical events must precede: (1) phosphorylation of I*κ*B*α* and I*κ*B*β* by the IKK complex, (2) ubiquitination of phosphorylated I*κ*B*α*, (3) proteasome-mediated degradation of I*κ*B*α* and I*κ*B*β*, (4) phosphorylation of NF-*κ*B dimer, (5) nuclear translocation of NF-*κ*B dimer, and (6) NF-*κ*B dimer-*κ*B DNA interaction (Figure 3).

## 12. Regulation of NF-*κ*B Activity via I*κ*B*α* Sumoylation

Signal-induced I*κ*B*α* phosphorylation and ubiquitination, followed by its proteolytic degradation, are not the only posttranslational modifications that target I*κ*B*α* and regulate NF-*κ*B activity in cells. Sumoylation is defined as process by which a small ubiquitin-like modifier (SUMO) (~20 kDa) is covalently attached to lysine residues on target proteins [195–197]. Similar to ubiquitination, the process of sumoylation involves three enzymatic events that proceed sequentially, ultimately culminating in SUMO conjugation to the protein substrate by forming an isopeptide bond between SUMO and the *ε*-amino group of a lysine side chain [198]. In 1998, Desterro and colleagues have reported for the first time the existence of a modified, slower migrating form of I*κ*B*α* [199]. This modified, slower migrating protein has been identified as an SUMO-1-modified I*κ*B*α* in several mammalian cells including human embryonic kidney HEK 293 cells, monkey COS-7, human T leukemic Jurkat cells, and HeLa cells [199]. Intriguingly, only a small fraction of total I*κ*B*α* protein was found to be modified by SUMO-1, and the degree of sumoylation varied depending on the cell type with 50% being the maximum proportion of sumoylated I*κ*B*α* of the total I*κ*B*α* pool [199]. Notably, nuclear localization of I*κ*B*α* was deemed necessary for its sumoylation [200]. Significantly, the sumoylated form of I*κ*B*α* was further shown to be highly resistant to signal-induced ubiquitination and subsequent proteasome-mediated degradation compared to unmodified I*κ*B*α* [199]. Desterro and colleagues went on to show that overexpression of SUMO-1 inhibits signal-induced activation of NF-*κ*B-dependent transcription. In a later study, Guo and colleagues have reported the identification and cloning of SUMO-4, which was proposed to conjugate with I*κ*B*α* leading to NF-*κ*B downregulation [201]. Very recently, SUMO-4-mediated downregulation of NF-*κ*B was shown to be dependent on modification of I*κ*B*α* by SUMO-4 [202]. Intriguingly, a *κ*B binding motif was also identified in SUMO-4 promoter, and mutagenesis of this motif interfered with NF-*κ*B-dependant transactivation of its target genes, suggesting a feedback loop mechanism by which SUMO-4 regulates NF-*κ*B activity [202].

Unlike ubiquitination, which requires phosphorylation at S^32^ and S^36^, phosphorylation at these sites interferes with I*κ*B*α* sumoylation [199], likely due to a conformational change that hinders SUMO-1 conjugation. Using site-directed mutagenesis, it was also shown that SUMO-1 conjugation requires K^21^ and K^22^ at the N-terminus of I*κ*B*α*; with K^21^ being the primary site for sumoylation [199]. Strikingly, K^21^ and K^22^ are the target residues for ubiquitination, providing a plausible explanation for the prolonged stability of sumoylated I*κ*B*α* compared to unmodified I*κ*B*α*. This observation also suggests that SUMO-1 and ubiquitin molecules compete for these residues to regulate I*κ*B*α* function, and thus, NF-*κ*B activity. Moreover, many hydrolases that can potentially cleave the bond between SUMO-1 and its targeted lysine residue on the substrate, hence known as desumoylating enzymes, have been identified [203–206]. Hence, controlling the balance between sumoylation and desumoylation of I*κ*B*α* may serve as a mechanism underlying the regulation of NF-*κ*B activity. Sumoylation of I*κ*B*α* may be a physiologically significant anti-inflammatory mechanism undertaken by cells to suppress lethal inflammatory responses via converting I*κ*B*α* proteins from their degradation-susceptible, unmodified form into a degradation-resistant, sumoylated one. Interestingly, in an in vitro study, it was demonstrated that epithelial cells exposed to increasing periods of hypoxia; a condition that triggers a wide range of inflammatory events, responded by increasing the proportion of SUMO-1-bound I*κ*B*α* and cAMP-response element-binding protein (CREB) [207]; a transcription factor that induced the expression of various proinflammatory cytokines. Consistently, induced hypoxia led to a significant increase in transcriptional expression of SUMO-1 [207]. Very recently, it was demonstrated that adenosine signaling mediates SUMO-1 modification of I*κ*B*α* during hypoxia [208]. Several studies have demonstrated a tight link between SUMO-4 polymorphism and susceptibility to type-I diabetes [201, 209, 210]. However, this correlation between SUMO-4 polymorphism and susceptibility to type-I diabetes seems to be more prevailing among Asian populations compared to Caucasians. Wang and colleagues have recently reviewed the correlation between SUMO-4 polymorphism and type-I diabetes, and they provided some insights to explain the discrepancy noted among different populations and the mechanisms through which SUMO4 contributes to the pathogenesis of type-I diabetes [211]. SUMO-4 polymorphism seems to have no correlation with susceptibility of other inflammatory conditions including Grave's disease [212], rheumatoid arthritis [213–215], and systemic lupus erythematosus [216]. Finally, it is conceivable that modulation of I*κ*B*α* sumoylation may be utilized as a mechanism to aggravate or alleviate the symptoms of various NF-*κ*B-driven inflammatory conditions.

It is important to mention that other proteins involved in NF-*κ*B signaling pathways are also subject to sumoylation. For example, sumoylation on K^277^ and K^309^ residues of NEMO by SUMO-1 conjugation has been shown to mediate NF-*κ*B activation by genotoxic stress [217]. In fact, regulation of NF-*κ*B activity by NEMO sumoylation occurs under a variety of other stress conditions including oxidative stress, ethanol exposure, heat shock, and electric shock [218]. Details regarding the mechanisms involved in NEMO sumoylation were revealed by a recent study demonstrating that NEMO sumoylation is mediated by protein inhibitor of activated STATy (PIASy), which seems to preferentially stimulate site-selective modification of NEMO by SUMO-1, but not SUMO-2 or SUMO-3, in vitro [219].

## 13. Cross-Talk between Glucocorticoid Receptor (GR) and NF-*κ*B Signaling

Although signal-induced posttranslational modification of I*κ*Bs by phosphorylation, ubiquitination, sumoylation, and proteolytic degradation serves as the central molecular mechanism by which NF-*κ*B signaling is regulated, several I*κ*B-independent mechanisms have been proposed as effective, alternative cellular events that are crucial in the regulation of NF-*κ*B activity. Such I*κ*B-independent mechanisms seem to be critical in the alternative, noncanonical NF-*κ*B signaling pathway and they occur via post-translational modification of various proteins, other than I*κ*Bs, that are critically involved in NF-*κ*B signaling. Like I*κ*Bs, some members of the Rel family of proteins can be subject to signal-induced post-translational modifications that ultimately lead to modulation of NF-*κ*B activity [134, 220, 221]. Signal-induced phosphorylation of RelA, which was first documented about fifteen years ago [67, 141], is by far the most extensively studied post-translational modification in the regulation of NF-*κ*B signaling (for review, refer to [134, 220, 221]). Similarly, post-translational modifications of RelB [222, 223], c-Rel [224–229], and p50 [67, 230] have also been documented as I*κ*B-independent regulatory mechanisms involved in NF-*κ*B signaling. 

Moreover, other I*κ*B-independent mechanisms involving a complex interplay between NF-*κ*B and other NF-*κ*B-unrelated proteins have been recently revealed. The glucocorticoid receptor (GR) is a prime example of such regulatory proteins that are critically implicated in the control of NF-*κ*B signaling pathways. GR is a hormone-dependent transcription factor belonging to the nuclear receptor superfamily, and it is critically involved in mediating the immunosuppressive functions of glucocorticoids by repressing the expression of major cytokines. Although the exact molecular mechanisms underlying the repression function of GR on NF-*κ*B activity are not fully understood, experimental evidence suggests that it is primarily the ligand-induced physical interaction between GR and DNA-bound NF-*κ*B subunits (RelA and p50) that ultimately inhibits NF-*κ*B activity [231–236]. The regulation of NF-*κ*B activity via the interaction between GR and NF-*κ*B subunits seems to be independent on GR DNA binding and homodimerization [236–238]. Although there is a consensus among researchers that the GR-mediated regulation of NF-*κ*B signaling takes place in the nucleus, it was proposed that ligand-induced activation of GR by glucocorticoids may regulate NF-*κ*B signaling in the cytoplasm by increasing I*κ*B*α* protein level in HeLa cells, monocytic cells, and T cells [239, 240]. However, induction of I*κ*B*α* seems to be cell type-specific mechanism underlying GR-mediated repression of NF-*κ*B activity since glucocorticoid treatment caused inhibition of NF-*κ*B activity without any detectable change in I*κ*B*α* expression in endothelial cells [241] or epithelial cells [242, 243]. In an in vitro study involving human pulmonary epithelial A549 cells, monkey COS-1 cells, and human breast cancer T47D cells, it was shown that GR-mediated inhibition of NF-*κ*B activity occurs via a dual mechanism involving both protein-protein interaction between GR and NF-*κ*B subunits and induction of I*κ*B*α* expression; with the former mechanism being predominant in NF-*κ*B regulation [244]. Moreover, it seems that GR-mediated repression of NF-*κ*B activity via I*κ*B*α* induction is not only cell type-specific, but it is also dependent on the type of ligand, the NF-*κ*B target gene, the presence of certain cofactors, the status of chromatin, and probably other conditions [221, 242, 245]. 

Noteworthy, other mechanisms underlying GR-mediated repression of NF-*κ*B activity have been proposed. For example, GR activation was shown to be associated with suppression of histone acetylase (HAT) activity via inhibited recruitment of large coactivator complexes containing HAT regulatory proteins such as CREB-binding protein (CBP) and p300 [246]. Moreover, GR activation was shown to cause induced expression of histone deacetylase 2 (HDAC2) accompanied by recruitment of GR to NF-*κ*B target genes leading to their transrepression [246]. It seems that the concentration of glucocorticoids is one determining factor in controlling the balance between GR-mediated suppression of HAT activity and induction of HDAC activity leading to modulated NF-*κ*B activity [246]. Interestingly, GR itself is subject to deacetylation by HDAC2, which leads to GR nuclear translocation and physical interaction with NF-*κ*B subunits [247]. It is also documented that induced histone methylation, rather than suppressed histone acetylation, may serve as a mechanism that underlies GR-mediated repression of NF-*κ*B functions [248, 249]. Additionally, experimental evidence revealed that attenuation of GR-mediated transrepression of its target genes is accompanied by recruitment of potent corepressors such as nuclear receptor corepressor (NCoR) and silencing mediator of retinoid and thyroid hormone receptor (SMRT) [250–253]. Furthermore, it was proposed that GR interferes with NF-*κ*B-dependent serine phosphorylation of the C-terminal domain of RNA polymerase II leading to suppressed expression of NF-*κ*B target genes [235]. These findings suggest that GR activation may repress NF-*κ*B activity without influencing NF-*κ*B DNA binding potential. Consistent with this proposal, treatment of asthmatic patients with inhaled glucocorticoids suppresses inflammation via inhibition of NF-*κ*B-mediated expression of inflammatory mediators with no detectable reduction in NF-*κ*B DNA binding ability [254]. Together, these findings strongly suggest that GR can modulate NF-*κ*B activity in the nucleus by regulating several key events including NF-*κ*B DNA binding, HAT and/or HDAC expression, coactivator(s) and/or corepressor(s) recruitment, as well as RNA polymerase II-induced transactivation of NF-*κ*B target genes. Finally, glucocorticoids may serve as inhibitors of NF-*κ*B activity via their ability to modulate the activity of proteins involved in the mitogen-activated protein kinase (MAPK) pathways including c-jun N-terminal kinase (JNK), p38, and MAP kinase phosphatase-1 (MKP-1), all of which can cross-talk and modulate NF-*κ*B activity, and thus, inflammatory responses [255–263].

## 14. AEBP1 Is a Multifunctional Protein

Adipocyte enhancer-binding protein-1 (AEBP1) is a ubiquitously expressed protein whose expression seems to be the highest in adipose tissue, liver, lung, spleen, and brain [264]. Recently, AEBP1 was shown to be abundantly expressed in primary macrophages as well as macrophage cell lines [265–267]. AEBP1 shares a remarkable amino acid sequence homology with two members of the regulatory carboxypeptidase family of enzymes, CPX1 and CPX2, all of which contain N-terminal discoidin-like domain (DLD) and homologous central carboxypeptidase (CP) domain [268]. Unlike CPX1 and CPX2, which are catalytically inactive [269, 270], AEBP1 functions as an active carboxypeptidase capable of catalyzing hydrolysis of arginine and lysine in hippuryl-arg and hippuryl-lys synthetic carboxypeptidase B (CPB) substrates, respectively [271, 272]. Studies from Ro's laboratory have demonstrated that deletion of residues 429 to 460 in the CP domain, which encompasses the active site, renders AEBP1 catalytically inactive [271]. Moreover, the carboxypeptidase activity of AEBP1 has been shown to be responsive to carboxypeptidase activators and inhibitors, and that DNA binding enhances AEBP1 hydrolytic activity [272], indicating that AEBP1 functions as an active carboxypeptidase. 

AEBP1 is highly expressed in preadipocytes, and its expression persists during the first stages of adipogenesis [271, 273]. However, AEBP1 levels drop dramatically as preadipocytes differentiate into mature adipocytes, and AEBP1 expression is completely abolished in terminally differentiated, nonproliferative adipocytes [264, 271, 273]. Because of the altered expression pattern of AEBP1 during adipogenesis, AEBP1 was suspected to play a negative regulatory role in adipose P2 (aP2) expression in preadipocytes. Indeed, in vitro studies have demonstrated that AEBP1 specifically binds adipocyte enhancer-1 (AE-1) DNA sequence [271], and transcriptionally represses aP2 in 3T3-L1 preadipocytes and other cell lines [271, 273, 274]. Transcriptional repression of aP2 by AEBP1 is physiologically significant since targeted over-expression of AEBP1 in adipose tissue causes diet-induced obesity in mice [275]. AEBP1 seems to induce massive obesity in mice with targeted, tissue-specific overexpression of AEBP1 (AEBP1^TG^ mice) by inducing adipocyte proliferation in vivo, leading to adipocyte hyperplasia in white adipose tissue [275]. In contrast, AEBP1-deficient mice (AEBP1^−/−^ mice) display 25% reduction in total body weight due to significantly reduced fat pads caused by enhanced apoptosis and impaired survival signal [276]. Indeed, AEBP1^TG^ preadipocytes display augmented proliferation [273, 275], while AEBP1^−/−^ preadipocytes exhibit a defective proliferative potential in vitro [276]. 

MAPK pathways are a network of serine/threonine kinases and dual-specificity kinases, whose function is implicated in various key biological processes in the cell including proliferation, inflammation, and tumorigenesis [277–280]. Kinases involved in MAPK pathways include JNK1/2, extracellular signal-regulated kinases 1 and 2 (ERK1/2), and other MAP kinases (e.g., MEK, MEKK, and MEKKK). In vitro and in vivo experimental studies revealed that AEBP1 physically interacts with ERK1/2 via its DLD [273]. This protein-protein interaction is critical for MAPK activity since it leads to protection of ERK1/2 from dephosphorylation by its specific phosphatase (MKP-3), leading to sustained activation of ERK1/2 [273]. AEBP1 inhibits differentiation of preadipocytes into mature adipocytes, thus impeding adipogenesis, by means of enhancing ERK1/2 activity in preadipocytes [273].

Recently, AEBP1 was shown to be a critical regulator of macrophage cholesterol homeostasis, foam cell formation, and macrophage inflammatory responsiveness [265]. In fact, AEBP1 was shown to manifest its proinflammatory effects by promoting NF-*κ*B activity via impeding the inhibitory function of I*κ*B*α* in macrophages, an event that seems to be dependent on AEBP1-I*κ*B*α* physical interaction [266]. Most recently, experimental evidence indicates that AEBP1 mediates LPS-induced foam cell formation by virtue of its ability to directly suppress peroxisome proliferator-activated receptor *γ* *1* *(*PPAR*γ*1) and liver X receptor *α* (LXR*α*) activity in macrophages, suggesting that AEBP1 may play a critical regulatory role in bacterial infection-induced atherosclerosis [267].

## 15. DLD Mediates AEBP1-I*κ*B*α* Interaction

**Figure 4:**
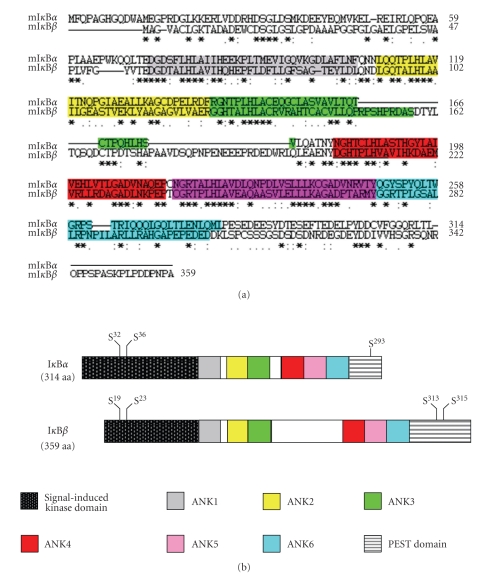
Amino Acid Sequence Comparison between  I*κ*B*α* and I*κ*B*β*. (a) Alignment of amino acid sequences of mouse I*κ*B*α* and I*κ*B*β* is shown. The six highly conserved ANK repeats in both proteins are highlighted in grey, yellow, green, red, pink, and blue, respectively. (b) A cartoon illustrating a comparison of the slightly different structural domain organizations of mouse I*κ*B*α* and I*κ*B*β* proteins. The signal-induced kinase domain, six ANK repeats, and PEST domain of each protein are shown.

The N-terminus of AEBP1 contains DLD that is remarkably homologous to discoidin, a lectin expressed in the slime mold *Dictyostelium discoideum* [281], and hence the name. In *Dictyostelium discoideum*, discoidin has been shown to be crucial for proper cell aggregation and migration [282] as well as protein-protein interaction [283, 284]. Indeed, DLD of AEBP1 was found to be required for protein-protein interaction between AEBP1 and MAPK [273]. Similarly, it was shown that AEBP1 is capable of physically interacting with I*κ*B*α* by means of its DLD, whose deletion eliminated AEBP1-I*κ*B*α* interaction [266]. It is worth mentioning that despite the structural similarities between I*κ*B*α* and I*κ*B*β*, co-immunoprecipitation experiments suggest that AEBP1 is capable of interacting with I*κ*B*α*, but not I*κ*B*β*, in macrophages [266]. Analysis of I*κ*B*α*-I*κ*B*β* amino acid sequence alignment reveals that there are three main structural differences between I*κ*B*α* and I*κ*B*β* (Figure 4). First, the first 12 amino acid residues in I*κ*B*α* are absent in I*κ*B*β*. Second, there is a 41-amino acid stretch located between the third and forth ANK repeat of I*κ*B*β* that is not present in I*κ*B*α*. Third, there is an 18-amino acid stretch at the C-terminus of I*κ*B*β* that is absent in I*κ*B*α*. Based on sequence analysis, it is conceivable that either the presence of the first 12 amino acid residues in I*κ*B*α* is required for interaction with AEBP1 or that the presence of the extra-amino acid stretches in I*κ*B*β* allows the formation of a tertiary structure that does not permit protein-protein interaction with AEBP1. It is also conceivable that the extra-amino acid stretches in I*κ*B*β* somehow mask the region, or domain, that is necessary for protein-protein interaction with AEBP1. We are currently attempting to map the exact region of I*κ*B*α* that mediates protein-protein interaction with AEBP1, which will shed more light on the differential ability of AEBP1 to regulate I*κ*B*α* and I*κ*B*β* functions. Further investigation of the AEBP1-interacting region of I*κ*B*α* will shed more light on how AEBP1 is capable of differentially regulating I*κ*B*α* and I*κ*B*β* functions in vivo. Although modulation of I*κ*B*α* expression has been previously shown to be a mechanism explaining altered NF-*κ*B activity [285–287], the data we have recently demonstrated is the first of its kind to propose a molecular mechanism behind modulated NF-*κ*B activity by which I*κ*B*α* protein stability is altered via protein-protein interaction and is independent of alterations in IKK complex kinetic activity [266].

Since about 50% of AEBP1 protein population exists in the nucleus [266, 288], and since newly synthesized I*κ*B*α* is known to translocate to the nucleus to bind DNA-bound NF-*κ*B dimers and resequester them into the cytosol [64], it is possible that AEBP1 and I*κ*B*α* interact in the nucleus. This is an interesting possibility since AEBP1-I*κ*B*α* interaction in the nucleus can interfere with the ability of nuclear I*κ*B*α* to bind to its target, DNA-bound NF-*κ*B dimers, leading to sustained NF-*κ*B-driven transactivation of target genes (e.g., proinflammatory genes). Thus, by virtue of its cytosolic/nuclear localization and its ability to bind I*κ*B*α*, it is reasonable to propose that AEBP1 can impede I*κ*B*α* inhibitory functions towards NF-*κ*B both at the cytosolic and nuclear levels. 

Noteworthy, DLD has been identified in several extracellular and intracellular proteins including discoidin domain receptor tyrosine kinase (DDR) [283], the blood coagulation cofactors V and VIII [289], milk-fat globule proteins [290], muskelin [284], retinoschisin [291], and developmental endothelial locus-1 (Del-1) [292]. It would be of great interest to assess whether such DLD-containing proteins have the potential to physically interact with I*κ*B*α*, as does AEBP1. Such assessment coupled with careful analysis of the amino acid variations among the DLD sequences of these proteins will assist in mapping the exact amino acid stretch within DLD that mediates interaction with I*κ*B*α*.

## 16. DLD Mediates AEBP1 Protein-Protein Interaction with Other Proteins

DLD has been suggested to mediate cell-cell adhesion [293], protein self-association [284], and protein-protein interaction [283, 284]. In fact, protein-protein interaction between AEBP1 and MAPK in the cytosol, which prolongs MAPK activation by protecting it from dephosphorylation by its specific phosphatase (MKP-3, also known as PYST1), has been shown to be mediated by DLD of AEBP1 [273]. Similarly, AEBP1 was shown to be capable of physically interacting with cytosolic I*κ*B*α* via its DLD, whose deletion eliminates AEBP1-I*κ*B*α* protein-protein interaction [266]. So, these findings further support a role of DLD in protein-protein interaction in mammalian systems. Furthermore, these findings underscore the importance of DLD in mediating very critical functions undertaken by AEBP1 to control key biological processes in the cell. Intriguingly, we propose that the presence of DLD creates a molecular competition between MAPK and I*κ*B*α* in the cytosol to bind to AEBP1. This proposal is interesting given that sustained MAPK activation and I*κ*B*α* proteolytic degradation followed by NF-*κ*B activation culminate in diverse biological outcomes in different cell types. Although the molecular mechanisms that signal AEBP1 to interact predominantly with MAPK or I*κ*B*α* are unknown, it is conceivable that AEBP1 can be utilized by the cell as an on/off switch to promote or inhibit MAPK and NF-*κ*B activities via balancing AEBP1 protein-protein interaction with MAPK and I*κ*B*α*.

## 17. AEBP1 and NF-*κ*B: A Positive Relationship

Since its initial identification by Sen and Baltimore about two decades ago [294], NF-*κ*B has been the focus of many researchers in an attempt to understand the various molecular mechanisms involved in inflammatory diseases and cancer. Modulation of NF-*κ*B activity can result in many abnormal cellular processes and diseases including asthma, arthritis, atherosclerosis, obesity, and various types of cancers [2–7]. Recently, we have provided experimental evidence establishing a positive relationship between AEBP1 expression and NF-*κ*B activity in macrophages [266]. Nuclear p65 protein level was shown to be barely detectable in AEBP1^−/−^ macrophages, compared to AEBP1^+/+^ counterparts [266]. Consistently, electrophoretic mobility gel shift assay clearly illustrates that ablation of AEBP1 expression in macrophages correlates with inhibited NF-*κ*B DNA-binding activity [266]. This positive relationship seems to be a consequence of a negative relationship between AEBP1 expression and I*κ*B*α* protein stability in macrophages. Interestingly, AEBP1 was shown to promote I*κ*B*α* phosphorylation followed by its proteolytic degradation, liberating the NF-*κ*B subunits, which translocate into the nucleus and become transcriptionally active [266]. Furthermore, this negative regulation imposed by AEBP1 on I*κ*B*α* function in the cytosol seems to be mediated by protein-protein interaction that requires DLD of AEBP1, as confirmed by co-immunoprecipitation analysis [266]. Consistent with the proposal that AEBP1-I*κ*B*α* protein-protein interaction, which is mediated by DLD, provokes destabilization of I*κ*B*α* shortening its half-life, the N-terminus deletion (ΔN) and carboxypeptidase (CP) mutant forms of AEBP1, which are devoid of DLD, have no influence on I*κ*B*α* protein stability, unlike AEBP1 derivatives retaining DLD [266]. Importantly, in contrast to the WT form of AEBP1, ΔN and CP mutant forms possess marginal or no upregulatory function towards NF-*κ*B activity [266], confirming that AEBP1-I*κ*B*α* interaction is a key biological event that is crucial for AEBP1-mediated I*κ*B*α*-induced degradation and subsequent NF-*κ*B up-regulation in macrophages. 

It is known that alteration of IKK*β* kinetic activity ultimately leads to modulation of I*κ*B*α* phosphorylation and proteolytic degradation [164]. Given that AEBP1 regulates I*κ*B*α* phosphorylation status and its proteolytic degradation, one may speculate that AEBP1 is capable of modulating I*κ*B*α* function in macrophages by means of altering the kinetic potential of IKK*β*. However, this possibility was ruled out by in vitro kinase assays demonstrating that IKK*β* kinetic activity against a bacterially expressed GST-I*κ*B*α* (aa 1-54) fusion protein is comparable in AEBP1^+/+^ and AEBP1^−/−^ macrophages under both basal and LPS-stimulatory conditions [266]. In addition, it was shown that AEBP1 is not a component of the IKK complex nor it influences the composition of the IKK complex in macrophages [266]. 

In a recent report, we have hypothesized that the positive regulatory role that AEBP1 imposes on NF-*κ*B activity may not be macrophage specific [266]. In fact, abrogation of NF-*κ*B activity has been shown to cause embryonic lethality due to liver apoptosis [25, 26, 164]. If AEBP1-mediated positive regulation of NF-*κ*B is a universal process that takes place in cells and tissues other than macrophages (e.g., liver), one would expect that AEBP1^−/−^ embryos may suffer from liver apoptosis that is life-threatening. Although NF-*κ*B activity has not been evaluated in AEBP1^−/−^ hepatocytes, it is fascinating that AEBP1^−/−^ mice suffer from about 50% embryonic lethality [276]. Hence, severe diminishment of NF-*κ*B activity, which can potentially lead to liver apoptosis, that is caused by AEBP1 deficiency may serve as a molecular mechanism underlying embryonic lethality in AEBP1^−/−^ mice. Interestingly, our recent findings reveal that the levels of the apoptotic markers p-STAT3 and cleaved caspase-3 are significantly higher in the livers of AEBP1^−/−^ mice compared to control mice (unpublished), suggesting that AEBP1 plays an antiapoptotic role in vivo. Consistent with its ability to promote NF-*κ*B activity in various cell types, preliminary findings suggest that AEBP1 also promotes NF-*κ*B activity in mammary gland tissue, in which NF-*κ*B activity is significantly enhanced and diminished in the mammary gland tissues obtained from mice that over-express and lack AEBP1, respectively (unpublished). In sum, AEBP1-mediated promotion of NF-*κ*B activity seems to be a regulatory event that occurs in various cells and tissues.

## 18. Differential Regulation of I*κ*B*α* and I*κ*B*β*: A Possible Role for AEBP1?

Despite their structural homology, individual I*κ*B proteins have distinctive structural features and they exhibit differential ability and preference to associate with and inhibit various combinations of NF-*κ*B dimers in the cytosol. For example, both I*κ*B*α* and I*κ*B*β* preferentially interact with and inhibit the activity of NF-*κ*B dimers containing p50, p65, and c-Rel [54, 123, 295], I*κ*B*γ* prefers p50 homodimers and p50/p65 heterodimers [296], I*κ*B*ε* prefers p65 and c-Rel homo- and heterodimers [297], and Bcl-3 prefers p50 and p52 homodimers [95, 298]; whereas p100 and p105 seem to bind to almost all possible dimer combinations of NF-*κ*B proteins [89, 299]. It is also known that different NF-*κ*B dimers display differential intrinsic preference with regard to DNA binding specificity [88, 300], and this DNA binding specificity confers distinct NF-*κ*B dimers a differential transactivation potential towards a diverse set of genes [301]. 

Additionally, I*κ*B*α* and I*κ*B*β* are known to be differentially regulated in various cell types and under several stimulatory conditions [143, 295]. Although both I*κ*B*α* and I*κ*B*β* become ubiquitinated upon phosphorylation, the two lysine residues (Lys^21^ and Lys^22^) at the N-terminus of I*κ*B*α* are required for ubiquitination; whereas the lysine residue (Lys^9^) at the N-terminus of I*κ*B*β* is not required for ubiquitination [143]. In addition, several studies have demonstrated that while I*κ*B*α* is subject to rapid degradation upon cell stimulation by various stimuli including LPS, IL-1*β*, TNF*α*, and PMA in most cell types, I*κ*B*β* degradation cannot be induced except by very few potent stimuli such as LPS and IL-1*β* in certain cell types and it tends to be a relatively slow process [147, 295]. However, other studies have shown that S^19^/S^23^-phosphorylated I*κ*B*β* is subject to degradation induced by PMA or TNF*α* treatment [143, 302]. The slower kinetics associated with I*κ*B*β* degradation has been suggested to be probably due to the slower rate of I*κ*B*β* phosphorylation by the IKK complex, which seems to favor I*κ*B*α* as a more efficient substrate [192]. So, depending on the potency of signals, I*κ*B*β* may or may not become subject to phosphorylation and subsequent degradation [10]. The differential ability of IKK*β* to phosphorylate I*κ*B*α* and I*κ*B*β* has been suggested as a mechanism to explain the differential proteolytic degradation kinetics of I*κ*B*α* and I*κ*B*α* [159]. Here, it can be argued that AEBP1, by virtue of its differential ability to interact with I*κ*B*α*, but not I*κ*B*β*, may play a determining role in making I*κ*B*α* more susceptible than I*κ*B*β* to signal-induced phosphorylation and subsequent degradation. Hence, AEBP1 physical interaction with I*κ*B*α*, but not I*κ*B*β*, has been proposed to serve as a mechanism that may elucidate the differential regulatory functions exhibited by these two molecules in vitro and in vivo [266]. 

Since the PEST domain plays a critical role in I*κ*B protein turnover/stability [52, 110, 143], it is arguable that the function of this domain is differentially regulated in I*κ*B*α* and I*κ*B*β*, thus leading to their differential regulation. However, studies have shown that deletion or mutations within the PEST domain confer resistance to signal-induced degradation for both I*κ*B*α* [110, 115, 117, 189–191] and I*κ*B*β* [143, 192–194]. In light of these results, understanding the role of PEST domain does not seem to help in explaining the differential regulation imposed on I*κ*B*α* and I*κ*B*β*. In addition, while the two N-terminal lysine residues (K^21^ and K^22^) of I*κ*B*α* are known to be ubiquitination sites that are required for signal-induced degradation of I*κ*B*α* [187, 188], the only N-terminal lysine residue (K^9^) in I*κ*B*β* does not seem to be an exclusive ubiquitination site, and its mutation has no effect on signal-induced degradation of I*κ*B*β* [143]. Moreover, it was shown that I*κ*B*β* is phosphorylated on Ser^19^ and Ser^23^ in unstimulated cells; whereas Ser^32^ and Ser^36^ phosphorylation in I*κ*B*α* is only signal induced [143]. 

In sum, the differential specificity of I*κ*B/NF-*κ*B interaction combined with the differential transactivation potential of different NF-*κ*B dimers may explain how differential regulation of distinct I*κ*B proteins can lead to differential regulation of NF-*κ*B dimer activity, and thus, differential expression control of discrete genes. However, due to the remarkable similarities between I*κ*B*α* and I*κ*B*β* in terms of their structure and NF-*κ*B dimer specificity, understanding the molecular mechanisms behind the differential regulatory functions undertaken by these two molecules in different cell types and under different conditions has proven to be a tremendous challenge, and so far, a crystal-clear explanation of such differential regulation of these two molecules is still lacking.

## 19. AEBP1-I*κ*B*α* Interaction Leads to I*κ*B*α*Degradation: Unknown Mechanism

To date, two pathways have been suggested as molecular mechanisms responsible for I*κ*B*α* proteolytic degradation. First, upon stimulation, I*κ*B*α* is thought to be degraded via a classical, signal-induced proteasome-dependent pathway that involves the 26S proteasome [170]. Second, in vitro studies using immature B cells have demonstrated that I*κ*B*α* can be subject to constitutive proteasome-independent, Ca^++^-dependent degradation under basal conditions [145]. It was also shown that constitutive phosphorylation of serine/threonine residues within the C-terminal PEST domain of I*κ*B*α* by CKII is required for I*κ*B*α* turnover [111–113]. Also, accumulation of free I*κ*B*α* in the cytosol triggers its rapid degradation through a phosphorylation, ubiquitination-independent proteasome-dependent pathway [144]. The exact molecular mechanism(s) underlying the regulatory role of AEBP1 towards I*κ*B*α* activity is not yet identified. However, we have questioned the molecular mechanisms by which AEBP1-I*κ*B*α* interaction leads to I*κ*B*α* phosphorylation and subsequent proteolytic degradation, and three speculative points regarding such molecular mechanisms were offered [266]. First, AEBP1-I*κ*B*α* interaction could cause a conformational change in the latter rendering it more susceptible to Ser^32^/Ser^36^ phosphorylation and degradation via the ubiquitination-dependent proteasome-dependent pathway. Second, I*κ*B*α*-bound AEBP1 could serve as a recruiting scaffold protein that facilitates recruitment of the constitutive proteasome-independent Ca^++^-dependent proteolytic or ubiquitination-independent proteasome-dependent machineries. Third, it is possible that AEBP1-bound I*κ*B*α* is more prone to constitutive phosphorylation on serine/threonine residues within the PEST domain, inducing its proteasome-dependent proteolytic degradation. Here, we speculate that AEBP1 may also serve as a “bridge” that brings I*κ*B*α* in proximity to IKK*α* */* *β* in the cytosol, forcing I*κ*B*α* phosphorylation and subsequent proteolytic degradation. In addition, it is possible that AEBP1 somehow enhances the catalytic activity of an “unknown” kinase that can potentially phosphorylate S^32^ and S^36^ in I*κ*B*α*. Moreover, one may speculate that AEBP1 interferes with an “unknown” phosphatase that exercises its catalytic activity on S^32^/S^36^-phosphorylated I*κ*B*α* in the cytosol. Finally, AEBP1 interaction with I*κ*B*α* may protect the latter from sumoylation, favoring its ubiquitination and subsequent proteolytic degradation. Examination of these possibilities may shed light on the exact molecular mechanism undertaken by AEBP1 to hamper I*κ*B*α* inhibitory function towards NF-*κ*B.

## 20. AEBP1-Mediated NF-*κ*B Upregulation Is Independent of PPAR*γ*1 and LXR*α*Modulation

Experimental evidence suggesting that PPAR*γ* *1* and LXR*α* play anti-inflammatory roles is overwhelming. PPAR*γ* and LXR ligands suppress inflammation by interfering with the NF-*κ*B, AP-1, and STAT signaling pathways [303–312]. PPAR*γ* *1* and LXR*α* repression by AEBP1 serves as a mechanism that satisfactorily explains the proinflammatory properties exhibited by AEBP1 in macrophages. Since AEBP1 represses PPAR*γ*1 and LXR*α* transcriptional activity in macrophages [265, 267], active PPAR*γ* *1* and LXR*α* interfere with NF-*κ*B activity [303, 304, 307, 313], and AEBP1 enhances NF-*κ*B activity, it is reasonable to suggest that PPAR*γ* *1* and LXR*α* transcriptional repression by AEBP1 may contribute to AEBP1-mediated NF-*κ*B up-regulation in macrophages. However, this effect may be negligible since deletion of DLD, which does not influence the ability of AEBP1 to repress PPAR*γ*1 or LXR*α* [265], completely eliminates the ability of AEBP1 to up-regulate NF-*κ*B activity [266]. In agreement, deletion of the C-terminus of AEBP1, which completely eliminates the ability of AEBP1 to repress PPAR*γ*1 or LXR*α* [265], does not interfere with the ability of AEBP1 to up-regulate NF-*κ*B activity [266]. Additionally, Glass and colleagues have shown that neither treatment of RAW 264.7 macrophages with PPAR*γ* ligand nor PPAR*γ* *1* overexpression in absence of its ligand had any anti-inflammatory effects [304]. Rather, PPAR*γ*-mediated anti-inflammatory effects are only observed when PPAR*γ* *1* is over-expressed and ligand activated [143]. Similarly, LXR*α*-mediated anti-inflammatory effects can only be observed in the presence of LXR ligands and under LPS-stimulatory conditions [307]. However, AEBP1 was shown to enhance NF-*κ*B activity in macrophages expressing endogenous PPAR*γ* *1* and LXR*α* in the absence of PPAR*γ* or LXR ligands under both basal and LPS-stimulatory conditions [266]. Collectively, we conclude that co-ordinate AEBP1-mediated I*κ*B*α* proteolytic degradation and subsequent NF-*κ*B up-regulation is independent of AEBP1-mediated PPAR*γ* *1* and LXR*α* repression in macrophages.

## 21. Potential Role of AEBP1 in Septic Shock Syndrome

Septic shock syndrome is a very serious medical condition that can lead to failure of many body organs, eventually causing death. Septic shock is caused by an exaggerated immune response against the LPS component of various Gram-negative bacteria via TLR signaling [320, 321]. Very recently, LPS was shown to significantly induce AEBP1 expression in macrophages, and that LPS-induced down-regulation of the pivotal anti-inflammatory mediators PPAR*γ*1 and LXR*α* is largely mediated by AEBP1 [267]. Given the role that AEBP1 plays in LPS signaling in macrophages, and given AEBP1 role in inducing macrophage proinflammatory responsiveness [265] via promoting NF-*κ*B activity in macrophages [266], it is conceivable that AEBP1^−/−^ mice may be resistant to LPS-induced septic shock and Gram-negative bacterial infection-induced atherosclerosis. In contrast, AEBP1^TG^ mice with targeted overexpression of AEBP1 in adipose tissue and macrophages [275] are expected to be more susceptible to LPS-induced septic shock and Gram-negative bacterial infection-induced atherosclerosis compared to their WT counterparts. It would be very intriguing to investigate the susceptibility of AEBP1^−/−^ and AEBP1^TG^ mice to develop septic shock syndrome upon administration of pathogenic Gram-negative bacteria such as *C. pneumonia*.

## 22. Other I*κ*B*α*-Interacting Proteins and Modulation of NF-*κ*B Activity

Recently, very few studies have proposed that protein-protein interactions involving I*κ*B*α* may serve as a molecular mechanism explaining the potential of some proteins to modulate NF-*κ*B activity in vitro and in vivo. Besides NF-*κ*B subunits, only a handful of proteins have been shown to physically interact with I*κ*B*α* using yeast two-hybrid system or co-immunoprecipitation experiments. The X protein of hepatitis B virus (HBV) has been shown to physically interact with I*κ*B*α*, but not I*κ*B*β*, and this interaction leads to sustained NF-*κ*B activation following TNF*α* treatment [314]. Mutagenesis analysis revealed that the 249-253 amino acid sequence towards the C-terminus of I*κ*B*α* is critical for protein-protein interaction between X protein and I*κ*B*α* [314]. Human *β*-arrestin2, a cytosolic protein that is expressed predominantly in the spleen and neuronal tissue, has been shown to directly interact with I*κ*B*α* leading to inhibited phosphorylation and degradation of I*κ*B*α*, ultimately leading to NF-*κ*B down-regulation [315]. The first 60 amino acids within the N-terminus of *β*-arrestin2 comprise the I*κ*B*α* interacting region, and the C-terminal domain (40 amino acids) of I*κ*B*α* contributes to the *β*-arrestin2 binding [315]. Using GST fusion protein pull-down assays, the poxvirus and zinc finger (POZ) domain at the N-terminus of FBI-1 (factor that binds to the inducer of short transcripts of human immunodeficiency virus-1), a ubiquitously expressed nuclear protein, has been shown to mediate protein-protein interaction between FBI-1 and I*κ*B*α* [316]. Additionally, *Chla*Dub1 is a deubiquitinating protease from *Chlamydia trachomatis* that has been shown to physically interact with I*κ*B*α*, leading to impaired ubiquitination and proteolytic degradation of I*κ*B*α* and blocked NF-*κ*B activation in transfected HeLa and HEK293N cells [317]. Similarly, human G protein-coupled receptor kinase 5 (GRK5), a protein that is highly expressed in the heart, placenta, lung and skeletal muscle, has been shown to interact with I*κ*B*α* in BAEC and HEK293 cells [318]. GRK5-I*κ*B*α* interaction has been shown to enhance nuclear accumulation of I*κ*B*α*, ultimately causing diminished NF-*κ*B-driven transcription and NF-*κ*B DNA binding [318]. The regulator of gene protein signaling homology domain of GRK5 (RH) and the N-terminal domain of I*κ*B*α* have been identified as the regions involved in GRK5-I*κ*B*α* interaction [318]. Finally, a yeast two-hybrid screen of a human library and co-immunoprecipitation experiments have recently revealed that N-terminal protease (N^pro^) of classical swine fever virus (CSFV), a protein that is localized in the cytosolic and nuclear compartments, physically interacts with I*κ*B*α* leading to transient nuclear accumulation of pI*κ*B*α* in the infected porcine kidney cell line PK-15 [319]. Yet, NF-*κ*B activation does not seem to be significantly affected in PK-15 cells that were stably transfected with N^pro^ [319]. It seems that the C-terminal (aa 213-317) region of I*κ*B*α* is essential for N^pro^-I*κ*B*α* interaction [319]. 

To date, however, AEBP1 is the only protein that was shown to be an interacting partner of I*κ*B*α* in macrophages, and AEBP1-I*κ*B*α* interaction seems to be physiologically significant with regard to NF-*κ*B transactivation and macrophage inflammatory responsiveness [266]. In an attempt to identify a consensus sequence that may be responsible of mediating protein-protein interaction between the I*κ*B*α*-interacting proteins mentioned above and I*κ*B*α*, the amino acid sequences of the I*κ*B*α*-interacting regions within these proteins were compared. Amino acid sequence analysis revealed that there is no obvious consensus sequence or considerable similarities within the identified I*κ*B*α*-interacting regions of AEBP1, FBI-1, *β*-arrestin2, and GRK5. Since the exact region of I*κ*B*α* that mediates physical interaction between I*κ*B*α* and these proteins is either different or unknown, it is difficult to search for other potential, yet unknown, I*κ*B*α*-interacting protein based on amino acid sequence similarities. The so-far identified I*κ*B*α*-interacting proteins and the domains/sequences involved in such interactions are outlined in Table 1.

## 23. Conclusion

NF-*κ*B signaling is critically involved in various biological processes that are crucial for cell growth and survival. The role that NF-*κ*B plays in inflammatory reactions cannot be underestimated. By virtue of its ability to act as a direct interacting partner of I*κ*B*α* via its DLD, AEBP1 exerts a potent upregulatory function toward NF-*κ*B activity in macrophages. Hence, AEBP1 manifests itself as a critical modulator of inflammatory responses. Finally, we anticipate that AEBP1 may serve as a likely molecular target towards the development of novel therapeutic strategies for the prevention or treatment of various inflammatory disorders such as atherosclerosis and septic shock syndrome.

## Figures and Tables

**Table 1:** I*κ*B*α*-Interacting Proteins and Impact on NF-*κ*B Activity.

I*κ*B*α*-Interacting Protein	Species	I*κ*B*α*-Interacting Region	Interaction Region of I*κ*B*α*	Impact on NF-*κ*B Activity	Reference
X protein	*Hepatitis B Virus*	Unknown	C-terminus (aa 249-253)	↑	[314]
*β*-arrestin2	*Homo sapiens*	N-terminus (aa 1-60)	C-terminus (aa 276-317)	↓	[315]
FBI-1	*Homo sapiens*	POZ domain (aa 24-131)	Unknown	↑	[316]
AEBP1	*Mus musculus*	DLD (aa 1-166)	Unknown	↑	[266]
*Chla*Dub1	*Chlamydia trachomatis*	Unknown	Unknown	↓	[317]
GRK5	*Homo sapiens*	RH domain (aa 50–176)	N-terminus (aa 1–58)	↓	[318]
N^pro^	*Classical Swine Fever Virus*	Unknown	C-terminus (aa 213–317)	↔	[319]

## Abbreviations

AE-1: Adipocyte enhancer-1
AEBP1: Adipocyte enhancer-binding protein-1
ANK: Ankyrin
AP-1: Activator protein-1
aP2: Adipose P2
ApoE: Apolipoprotein E
cAMP: Cyclic adenosine monophosphate
CBP: CREB-binding protein
CKII: Casein kinase II
COX-2: Cyclooxygenase 2
CP: Carboxypeptidase
CPB: Carboxypeptidase B
DLD: Discoidin-like domain
ELKS: Glutamic acid, leucine, lysine, and serine-rich protein
ERK: Extracellular signal-regulated kinase
FBI-1: Factor that binds to the inducer of short transcripts of HIV-1
GR: Glucocorticoid receptor
GRK: G protein-coupled receptor kinase 5
GST: Glutathione S transferase
HAT: Histone acetylase
HDAC: Histone deacetylase
ICAM-1: Intracellular adhesion molecule-1
IL: Interleukin
IFN*γ*: Interferon *γ*
I*κ*B: Inhibitor of NF-*κ*B
IKK: I*κ*B kinase
iNOS: Inducible nitric oxide synthase
IRAK: Interleukin 1 receptor-associated kinase
JNK: C-jun N-terminal kinase
LPS: Lipopolysaccharide
LXR*α*: Liver X receptor *α*
MAPK: Mitogen-activated protein kinase
MCP-1: Monocyte chemoattractant protein-1
MEKK: MEK kinase (also designated as MAPKKK)
MKP: MAP kinase phosphatase
MMP: Matrix metalloproteinase
NCoR: Nuclear receptor co-repressor
NEMO: NF-*κ*B essential modulator
NES: Nuclear export signal
NF-*κ*B: Nuclear factor kappa B
NIK: NF-*κ*B inducing kinase
NLS: Nuclear localization signal
N^pro^: N-terminal protease
oxLDL: Oxidized low density lipoprotein
PEST: Proline-glutamate/aspartate-serine-threonine
PIASy: Protein inhibitor of activated STATy
PKA: Protein kinase A
PKB/AKT: Protein kinase B
PKC: Protein kinase C
PMA: Phorbol-12-myristate-13-acetate
POZ: Poxvirus and zinc finger domain
PPAR*γ**1*: Peroxisome proliferator-activated receptor *γ* *1*
RHD: Rel homology domain
RIP: Receptor interacting protein
SMRT: Silencing mediator of retinoid and thyroid hormone receptor
STAT: Signal transducer and activator of transcription
SUMO: Small ubiquitin-like modifier
TAD: Transactivation domain
TNF*α*: Tumor necrosis factor *α*
TRADD: TNF receptor-associated death domain
TRAF: TNFR associated factor
VCAM-1: Vascular cell adhesion molecule-1
WT: Wild type
